# Self-rated mental health and socio-economic background: a study of adolescents in Sweden

**DOI:** 10.1186/1471-2458-14-394

**Published:** 2014-04-23

**Authors:** Katrin Hutton, Maria Nyholm, Jens M Nygren, Petra Svedberg

**Affiliations:** 1 Affecta psychiatric out-patients clinic, Sperlingsgatan 5, 302 48 Halmstad, Sweden; 2 School of Social and Health Sciences, Halmstad University, SE 301 18 Halmstad, Sweden

**Keywords:** Adolescents, Self-rated mental health, Socio-economic status, Family affluence scale

## Abstract

**Background:**

Adolescents’ mental health is a major public health issue. Previous research has shown that socio-economic factors contribute to the health status of adolescents. The present study explores the association between socio-economic status and self-rated mental health among adolescents.

**Methods:**

Cross sectional data from the Halmstad Youth Quality of Life cohort was collected in a town in Sweden. In all, 948 adolescents (11–13 younger age group and 14–16 older age group) participated. Information on self-rated mental health was collected from the subscale Psychological functioning in the Minneapolis Manchester Quality of Life instrument. The items were summarized into a total score and dichotomized by the mean. Indicators measuring socio-economic status (SES) were collected in a questionnaire using the Family Affluence Scale (FAS) and additional factors regarding parents’ marital status and migration were added. Logistic models were used to analyze the data.

**Results:**

Girls were more likely to rate their mental health below the mean compared to boys. With regard to FAS (high, medium, low), there was a significantly increased risk of self-rated mental health below the mean among younger boys in the medium FAS score OR; 2.68 (95% CI 1.35;5.33) and among older boys in the low FAS score OR; 2.37 (1.02;5.52) compared to boys in the high FAS score. No such trend was seen among girls. For younger girls there was a significant protective association between having parents born abroad and self-rated mental health below mean OR: 0.47 (0.24;0.91).

**Conclusions:**

A complex pattern of associations between SES and self-rated mental health, divergent between age and gender groups, was shown. The total FAS score was only associated with boys’ self-rated mental health in both age groups, whereas parents’ migratory status influenced only the girls’ self-rated mental health. Because of the different association for girls’ and boys’ self-rated mental health and SES, other factors than SES should also be considered when investigating and exploring the mental health of adolescents in affluent communities.

## Background

Deterioration of mental health among adolescents is considered to be a substantial public health concern, motivating preventative interventions [1]. It is argued that interventions designed to reduce health inequalities early in childhood may help move children onto healthier lives, with the hope of maximising health, including mental health, outcomes across the life course [2].

Studies have shown the impact of socio-economic status (SES) on present and future mental health [3,4]. In relation to mental health, studies have focused on psychological distress (including symptoms of depression, anxiety and stress) or mental health in the form of depression and bipolar disorder. An association between low SES and self-reported mental health in adults, including psychological distress, has been found in several studies [5-10]. However, the link between low SES among adolescents and mental health in later life is less clear [3,4], as is the association between SES and youth mental health [3,11,12].

Income, education and occupation are common indicators used as a basis for measuring adult SES [13,14]. However, measuring SES in adolescents is difficult as adolescents may be unwilling or unable to specify their parents’ economic status and educational achievement, leading to high levels of missing data [13,15]. The Family Affluence Scale (FAS) is a widely used standardized instrument for measuring adolescent SES [13]. It comprises four items; car ownership, own bedroom, family holidays and computer ownership; it is therefore a resource-based indicator of SES. Factors such as migration and marital status are also often included in studies regarding health and SES [16]. These factors are important determinants of socio-economic resources available to adolescents [17]. Experience of separation or divorce in the family has been found to affect the health of adolescents in a negative way [18,19]. However, the research has shown divergent results of the effect of living in a single-parent household [20-24]. An increased prevalence in mental and psychological health problems among migrants has also been shown [25-27].

In the present study, we explore the association between SES and self-rated mental health among Swedish adolescents between 11–16 years old, using FAS and additional factors regarding parents’ status as a measurement of SES.

## Methods

### Subjects and data collection

Halmstad is a municipality in south western Sweden with a high ranking in national comparisons of affluence [28]. At the time when this study took place, autumn 2011, it had a population of 92 000 inhabitants and a local economy characterized by small and medium-sized companies. Approximately 14% of the population were foreign-born (15% country average), the unemployment rate was 7% (slightly higher than the country average 6.5%), 8% of the inhabitants received sickness benefits or equivalent (compared to the country average 7%). Approximately 4% of households received welfare benefits compared to the country average 6.5% [29]. There were 34 public and four private schools in total. Halmstad is the main town with approximately 62 000 inhabitants.

On the basis of being centrally located in Halmstad and having more than 100 pupils (aged 11–13 years and 14–16 years), seven public schools were selected and invited to participate in the study. A total of 50 classes were invited. One class in the older age group opted out of participation. A sample of 24 classes with pupils in the younger age group (n = 536 pupils) and 25 classes with pupils in the older age group (n = 576 pupils) were included. 948 respondents (467 pupils in the younger age group and 481 in the older age group) agreed to participate and completed the questionnaires (response rate 87% and 84% respectively). In the younger age group and in the older age group, the questionnaires of 58 and 60 adolescents respectively, were excluded due to missing data. The final sample consisted of 830 adolescents.

Adolescents answered self-report questionnaires consisting of the Minneapolis Manchester Quality of Life instrument, (MMQL), the FAS scale and questions regarding parents’ migration and marital status. Adolescents of 11–13 years were defined as the younger age group and those of 14–16 years were defined as the older age group. The principal at each school approved participation. Before the study was carried out the school distributed a written information to children and their parents about the purpose of the research, that the participation was voluntary and if the children or the parents declined to participate, they could decide not to fill in the questionnaire without having to explain why. Questionnaires were distributed in each class following a brief introduction by the research team. Participation was voluntary and children who waived participation could return blank questionnaires. Completed questionnaires were returned by each respondent directly and collected by the authors, except for two schools where teachers distributed and collected the questionnaires in return envelopes for each class.

### MMQL and self-rated mental health

MMQL is a self-assessment instrument available in two age appropriate versions, the MMQL-Youth form for children aged 8–12 years [30] and the MMQL-Adolescent form for children aged 13–20 years old [31]. Both versions have been designed to cover the same areas of self-rated health related quality of life through age-specific questions [30,31]. The MMQL-Youth form consists of four quality of life domains; physical symptoms, physical functioning, psychological functioning and outlook on life/family dynamics (divided into 32 items). The MMQL-Adolescent Form consists of seven quality of life domains; physical functioning, cognitive functioning, psychological functioning, body image, social functioning, intimate relations and outlook on life (divided into 45 items). In this study we only used the subscale psychological functioning to determine self-rated mental health (MMQL-PF). The reliability and validity of MMQL has been assessed in a study of healthy children and children with cancer in the USA [30,31]. Both instruments have good psychometric characteristics and are available in versions translated and validated in a Swedish context [32]. The subscale psychological functioning contains items about how often you; feel sad, angry, lonely and afraid; have anxiety for dying, your health, in general and; don’t feel as good as others. In the older age group three more questions were asked about how often you feel; anxious and nervous, strong and healthy or tired during the day. The responses in each item are based on the number of points in the scale; “Never” = 5; “Seldom” = 4, “Sometimes” = 3, “Mostly” = 2 and “Always” = 1. All items were summarized into a total score in the different age- and gender-groups and further dichotomized by the mean. Below mean was categorized as “1” and above mean as “0”.

### Socio-economic variables

#### Family affluence scale

Using the FAS-scale, adolescents’ SES was characterized by parental ownership of cars (Does your family own a car, van, or a truck? (0 = no, 1 = yes, one, 2 = yes, two or more), sharing or not sharing a room (Do you have your own room? (0 = no, 1 = yes), number of holidays per year (During the past 12 months, how many times did you travel away on holiday with your family? (0 = not at all, 1 = once, 2 = twice, 3 = more than twice), having computers at home (How many computers does your family own? (0 = none, 1 = one, 2 = two, 3 = more than two) [13]. A composite FAS score was calculated by adding the responses for the four items ranging from 0–9 [13].

FAS was explored in three different ways. Firstly, each item was analysed in association with self-rated mental health. Secondly, the FAS scale was analysed using the whole range of the scale (i.e. 0–1, 2, 3, 4, 5, 6, 7, 8–9) with 8–9 as a reference. Thirdly, the composite FAS score, in order to reflect the relatively wealthy municipality, was recoded into low (0–5), medium (6–7) and high (8–9) with “high” as reference.

#### Additional factors regarding parents’ status

Parents’ marital status was measured by the question “Are your parents divorced?” The answers were coded as 1 (=yes) and 0 (=no) using “not divorced” as reference. Migration of parents was measured by the question “Was your father born in Sweden?” and “Was your mother born in Sweden?” The answers were added together and then coded as both parents born in Sweden (=0) or one or two parents born outside Sweden (=1). The category “not born abroad” was used as reference.

### Statistical methods

The statistical analyses were carried out using SPSS statistics version 20.0 (IBM, New York, USA). Continuous variables were expressed as mean and standard deviation (SD). Chi-square and independent student t-tests were conducted to compare MMQL-PF, FAS, parents’ marital status (not divorced and divorced) and parents’ migration status (parents not born in Sweden and parents born in Sweden) between gender and age. Gender and age groups were analysed separately, i.e., the reference point (mean) was specific for each age and gender group. Significance was assumed at p < 0.05. Logistic regression was used in the analyses. Results were reported as odds ratio (OR) with 95% confidence intervals (CI). Adolescence school affiliation was adjusted for by including dummy variables for each school in the regression model [33]. The association between self-rated mental health (MMQL-PF), FAS and parents’ migration and marital status were analysed. Scores of MMQL-PF below mean represent worse self-rated mental health and scores above mean represent a higher self-rated mental health.

### Ethical consideration

Permission for the study was obtained from the local ethics committee at Halmstad University (Dnr 90-2011-2863). The participants were guaranteed anonymity, were informed that participation was voluntary and told that they did not need to fill in the questionnaire if they did not want to or if their parents objected.

## Results

In general, girls rated their mental health significantly lower compared to boys, both in the younger age group (p = 0.002) and in the older age group (p < 0.001) (Table 1). There were no statistical differences between boys’ and girls’ FAS scores, parents’ marital status and parents’ migration status.

**Table 1:** Characteristics of respondents, distribution of self-rated mental health, family affluence scale, parents marital status, migration

		***Younger age group***					***Older age group***				
**Characteristics**	**Variable**	**Boys (n = 207)**		**Girls (n = 207)**			**Boys (n = 232)**		**Girls (n = 199)**		
		**Mean**	**SD**	**Mean**	**SD**	**p value**	**Mean**	**SD**	**Mean**	**SD**	**p value**
*Self-rated mental health*		4.17	0.49	4.0	0.59	**0.02**	4.01	0.57	3.81	0.56	**<0.001**
		**n**	**%**	**n**	**%**	**p value**	**n**	**%**	**n**	**%**	**p value**
*Family affluence scale (FAS)*											
Does your family own a car?	No to one car	108	52.2	115	55.6	0.116	117	50.4	104	52.3	0.705
	Two and more	99	47.8	92	44.4		115	49.6	95	47.7	
Do you have your own bedroom for yourself?	No	30	14.5	28	13.5	0.777	19	8.2	24	12.1	0.181
	Yes	177	85.5	179	86.5		213	91.8	175	87.9	
During the past 12 months, how many times	Not at all to once	106	51.2	106	51.2	0.999	131	56.5	112	56.3	0.969
did you travel away on holiday with your family?	Twice to more than twice	101	48.8	101	48.8		101	43.5	87	43.7	
How many computers does	None to one	22	10.6	24	11.6	0.754	15	6.5	12	6.0	0.852
your family own?	Two to more than two	185	89.4	183	88.4		217	93.5	187	94.0	
*FAS score*	Low (FAS 0–5)	63	28.3	70	32.3	0.385	50	20.4	54	25.2	0.483
	Medium (FAS 6–7)	93	41.7	91	41.9		129	52.7	105	49.1	
	High (FAS 8–9)	67	30.0	56	25.8		66	26.9	55	25.7	
*Marital status*	Not divorced parents	159	68.7	156	68.7	0.666	168	67.5	152	69.1	0.733
	Divorced parents	69	30.3	71	31.3		81	32.5	68	30.9	
*Migration parents place at birth*	No parents born abroad	149	63.4	144	62.3	0.391	157	62.5	145	65.0	0.483
	Parents born abroad	86	36.6	87	37.7		94	37.5	78	35.0	

p < 0.05.

### Self-rated mental health and family affluence scale

The FAS items were significantly and differently associated to gender and age (Table 2). For girls, there was only an association with FAS and self-rated mental health at item level. In the younger age group, there was a significant protective association between self-rated mental health below mean and not having an own bedroom OR: 0.23 (95% CL: 0.08; 0.61). Having none to one holiday with your family was associated with an increased risk of rating below the mean compared to having two or more holidays for girls in the younger age group OR; 1.90 (95% Cl 1.08; 3.36) and boys in the older age group OR; 1.96 (1.11;3.45).

**Table 2:** Odds ratio (OR ) with 95% confidence interval (95%CI) for self-rated mental health below the mean by family affluence

	***Younger aged group***				***Older age group***			
	**Boys**		**Girls**		**Boys**		**Girls**	
	**OR**	**95% Cl**	**OR**	**95% Cl**	**OR**	**95% Cl**	**OR**	**95% Cl**
*Family affluence scale (FAS)*								
*Items*								
*Does your family own a car?*								
Two and more	1.0	(Reference)	1.0	(Reference)	1.0	(Reference)	1.0	(Reference)
No to one car	1.46	(0.82;2.59)	1.27	(0.63;2.31)	1.16	(0.66;2.05)	1.04	(0.55;1.95)
*Do you have your own bedroom for yourself?*								
Yes	1.0	(Reference)	1.0	(Reference)	1.0	(Reference)	1.0	(Reference)
No	0.72	(0.32;1.63)	0.23	(0.08;0.61)	1.14	(0.41;3.20)	1.09	(0.42;2.86)
*During the past 12 months, how many times*								
*did you travel away on holiday with your family?*								
Twice to more than twice	1.0	(Reference)	1.0	(Reference)	1.0	(Reference)	1.0	(Reference)
Not at all to once	1.47	(0.84;2.55)	1.90	(1.08;3.36)	1.96	(1.11;3.45)	0.92	(0.50;1.69)
*How many computers does your family own?*								
Two to more than two	1.0	(Reference)	1.0	(Reference)	1.0	(Reference)	1.0	(Reference)
None to one	0.88	(0.36;2.19)	0.60	(0.25;1.47)	1.77	(0.58;5.40)	0.62	(0.18;2.13)
Numbers of FAS score:								
9–8	1.0	(Reference)	1.0	(Reference)	1.0	(Reference)	1.0	(Reference)
7	3.58	(1.62;7.92)	0.71	(0.31;1.61)	1.28	(0.57;2.84)	1.17	(0.56;2.63)
6	1.78	(0.75;4.24)	0.79	(0.34;1.82)	1.74	(0.81;3.71)	0.90	(0.37;2.19)
5	1.68	(0.67;4.22)	1.51	(0.59;3.87)	2.41	(0.85;6.85)	0.45	(0.17;1.21)
4	1.75	(0.61;5.00)	0.79	(0.30;2.10)	1.26	(0.36;4.39)	0.36	(0.08;1.72)
1–3	1.87	(0.47;7.36)	0.70	(0.16; 3.08)	5.19	(0.90;29.71)	2.97	(0.49;18.16)
*FAS score categories*								
High (score range: 8–9)	1.0	(Reference)	1.0	(Reference)	1.0	(Reference)	1.0	(Reference)
Medium (score range: 6–7)	2.68	(1.35;5.33)	0.75	(0.37;1.52)	1.58	(0.80; 3.14)	1.04	(0.51;2.15)
Low (score range: 0–5)	1.74	(0.82;3.69)	1.06	(0.50;2.27)	2.37	(1.02; 5.52)	0.56	(0.24;1.34)

Secondly, the FAS scale was used comparing the highest score (8–9 FAS score) to 7, 6, 5, 4, 0–3 FAS score. Boys in the younger age group were likely to significantly rate their mental health below mean when having FAS score 7 compared to boys with a higher score (11–13 years, OR; 3.58 (95% CI 1.62; 7.92)). No such trend was seen among girls or older boys. Thirdly, in comparison, FAS was grouped into scores high, medium, and low. There was a significantly increased risk of self-rated mental health below the mean among younger boys in the medium FAS score OR; 2.68 (95% CI 1.35;5.33) compared to boys in the high FAS score. For older boys, a significantly increased risk was apparent in the low FAS score OR; 2.37 (95% CI 1.02;5.52) compared to boys in the high FAS score. There was no significant association between self-rated mental health and the total FAS score in girls.

### Self-rated mental health, parents marital status and parents migration

Table 3 presents adolescents’ self-rated mental health and its relationship to parents’ migration and marital status. In the younger age group there was a significant protective association for girls with parents born abroad and self-rated mental health OR: 0.47 (95% CI 0.24;0.91). No such trend was seen in the older age group for boys or girls. There was no association for any age group when having divorced parents.

**Table 3:** Odds ratio (OR ) with 95% confidence interval (95%CI) for self-rated mental health below the mean by parental migration and parental marital status

	***Younger aged group***				***Older age group***			
	**Boys**		**Girls**		**Boys**		**Girls**	
	**OR**	**95% Cl**	**OR**	**95% Cl**	**OR**	**95% Cl**	**OR**	**95% Cl**
*Parents migration*								
No parents born abroad	1.0	(Reference)	1.0	(Reference)	1.0	(Reference)	1.0	(Reference)
Parents born abroad	0.92	(0.49;1.72)	0.47	(0.24;0.91)	1.48	(0.79;2.77)	0.63	(0.31;1.28)
*Parents marital status*								
Not divorced	1.0	(Reference)	1.0	(Reference)	1.0	(Reference)	1.0	(Reference)
Divorced	1.20	(0.63;2.29)	1.18	(0.64;2.19)	1.15	(0.64;2.08)	1.50	(0.79;2.88)

## Discussion

In this study, we utilised two assessments of SES, both from the adolescents’ perspective through FAS and through additional factors regarding parents’ status. An association between self-rated mental health and the total FAS score was only apparent for boys in both age groups. Parents’ marital status did not seem to indicate an increased risk for self-rated mental health below mean for either gender- or age group. A positive association was seen for girls’ self-rated mental health in the older age group having parents born abroad.

Boys in both age groups seemed to exhibit a higher risk of self-rated mental health below mean in association to FAS. This result was evident in all different analyses of FAS. There was a lack of association between the total FAS score and girls’ self-rated mental health, however, at item level (having an own bedroom and number of holidays) there were associations in opposing directions. These item-level findings might be factors that could influence the total FAS score for girls and the opposing association between the different items might affect the total FAS score for girls. The opposing associations for individual items may cancel each other out resulting in no association between the total score and girls’ mental health. Other research in Nordic countries has similar findings [34], suggesting that girls’ lower self-rated health may be affected by different parameters compared to boys [12,34,35].

The theory of equalisation in youth suggests limited association between SES and self-rated mental health. It proposes that adolescence is characterised by a relative equality of health due to changes in social class patterns during transition from childhood, youth and subsequent adulthood [5]. The diminished effect of social class on self-rated mental health has been suggested to relate to other influences than SES and background, such as school and peer groups. In the same strain, the subjective social status (SSS) has been proposed as a predictor of health [36]. The theory of SSS suggests that health of individuals is related to the individuals’ perception of his/her relative wealth and position in the social hierarchy. Some groups, more than others, seem to rely more on relative comparisons within the group that can be difficult to measure with traditional and absolute SES measures [36]. Although no data was collected on SSS in our study, it is possible that SSS explains the different results of self-rated mental health between older and younger boys as the importance of SSS might change with age, however the importance of SSS in this regard has to be evaluated in future studies.

Parents’ migration status in our study showed an impact only on girls’ self-rated mental health. Other studies have shown varied results [26,37-43]. A review on the mental health in first and second-generation immigrant boys and girls showed no unequivocal results, both higher and lower levels of mental health problems were found [39]. Worse scores of health outcomes have been found for girls with immigrant parents, interpreted as illustrative of the difficulties for adolescent girls in adjusting and accommodating two cultures [38]. However, other researchers propose that adapting to a new culture and society is easier for second-generation immigrant girls, enabling them to embrace new opportunities in their adopted country, leading to improved mental health [44]. In our study, having parents born abroad acted as a protective factor for self-rated mental health among younger girls. For older girls, or boys of any age, no positive or negative results were seen.

Regarding the other aspect of parents’ status we investigated, research shows that children of divorced parents are affected negatively [37,41]. Our study could not show any association between having divorced parents and a risk of self-rated mental health below mean. It might be important to explore whether it is the family structure *per se*, or the characteristics of households and community contexts that affect the mental health of adolescents [42]. The few associations in this study between parents’ migration, as well as marital status, and adolescents’ self-rated mental health might indicate that these parental factors are of less importance and might rather be considered mediators of SES as previously suggested [26].

### Strengths and limitations

The cross-sectional design of the study implies a weakness since the causal mechanisms cannot be inferred, nor can the results from this study population be generalized. The primary sampling unit was the school. The collection of data was not through traditional random cluster sampling. The schools were included based on particular characteristics such as having a size large enough to avoid identification of students and proximity to central Halmstad. The homogeneity of the sample influenced our choice not to use multi-level models. This might be a limitation, however, we did not foresee that the results would have changed significantly. The study was based upon self-reported data, which strengthens the results as they are based on children’s own perceptions of their mental health.

This study used FAS as SES indicator. The FAS scale primarily measures socio-economic position from a material point of view and is recognized as a good measurement of adolescent SES [45]. The possibility of social bias, through cultural and structural surroundings, cannot be excluded in the FAS reports or in the self-reported mental health score. FAS is a culturally and time-sensitive tool and needs recurring evaluation with consideration of material trends and opportunities [13]. For example, present use and possession of smartphones and tablets, in fact, provides adolescents with constant availability of digital social interaction, information flows and tools. However, this is not specified or well accounted for in the original design of the FAS scale and might have influenced our results. The lowest category of FAS was categorized as 0–5 items compared to other studies where the lowest category has been defined as 0–3 items, due to the fact that Halmstad is a comparatively wealthy town [13]. The chosen cut-off point for MMQL-PF was the mean. The reason for this was the relatively high rating scores of the scale and a possible lack of power with a more extreme cut-off point.

We also chose to add the factors parents’ migration and marital status to reflect the multiple and contextual aspects of SES. However, the present marital status does not necessarily reflect the current family structure where parents might be re-married or cohabitating, hence generally providing more resources and higher SES than a single-parent household. We chose this question considering the age structure of the sample. However, retrospectively, a more poignant question could have focused on current living conditions and family structure rather than marriage.

## Conclusions

This study shows a complex pattern of associations between SES and self-rated mental health. The results diverged between age and gender groups. The main conclusion is that the total FAS score was only associated with boys self-rated mental health below mean in both age groups, whereas parents’ migratory status influenced only the girls’ self-rated mental health. However, since the association for girls’ and boys’ self-rated mental health and SES differed, other factors than SES should also be considered when investigating and exploring the mental health of adolescents in affluent communities.

## Competing interests

The authors declare that they have no competing interests.

## Authors’ contributions

The study was originally initiated and designed by PS and JN. The data collection was done by PS and JN. MN has provided the statistical analysis. The manuscript was drafted by KH and MN with supervision of PS and JN. All of the authors have contributed to this study with interpretation of the results as well as to the drafting of the final version. Critical revisions for significant intellectual content were made by all authors. All authors read and approved the final manuscript.

## Pre-publication history

The pre-publication history for this paper can be accessed here:

http://www.biomedcentral.com/1471-2458/14/394/prepub

## References

[B1] SBU – Swedish Council on Health Technology Assessment Methods to Prevent Mental ill-Health in Children 2010 Stockholm http://www.sbu.se/sv/Publicerat/Gul/Program-for-att-forebygga-psykisk-ohalsa-hos-barn/28876792

[B2] Patel V Flisher AJ Hetrick S McGorry P Mental health of young people: a global public-health challenge Lancet 2007 14 1302 1313 17434406 10.1016/S0140-6736(07)60368-7

[B3] West P Health inequalities in the early years: is there equalization in youth? Soc Sci Med 1997 44 833 858 10.1016/S0277-9536(96)00188-8 9080566

[B4] Power C Stansfeld SA Matthews S Manor O Hope S Childhood and adulthood risk factors for socio-economic differentials in psychological distress: evidence from the 1958 British birth cohort Soc Sci Med 2002 55 1989 2004 10.1016/S0277-9536(01)00325-2 12406466

[B5] Cheung YB Khoo KS Karlberg J Machin D Association between psychological symptoms in adults and growth in early life: longitudinal follow up study BMJ 2002 5 749 12364303 10.1136/bmj.325.7367.749 PMC128376

[B6] Torsheim T Currie C Boyce W Kalnins I Overpeck M Haugland S Material deprivation and self-rated health: a multilevel study of adolescents from 22 European and North American countries Soc Sci Med 2004 59 1 12 10.1016/j.socscimed.2003.09.032 15087138

[B7] Torsheim T Ravens-Sieberer U Hetland J Välimaa R Danielson M Overpeck M Cross-national variation of gender differences in adolescent subjective health in Europe and North America Soc Sci Med 2006 62 815 827 10.1016/j.socscimed.2005.06.047 16098649

[B8] Richter M Erhart M Vereecken CA Zambon A Boyce W Gabhainn SN The role of behavioural factors in explaining socio-economic differences in adolescent health: a multilevel study in 33 countries Soc Sci Med 2009 69 396 403 10.1016/j.socscimed.2009.05.023 19540029

[B9] Poulton R Caspi A Milne BJ Thomson WM Taylor A Sears MR Moffitt TE Association between children’s experience of socioeconomic disadvantage and adult health: a life- course study Lancet 2002 360 1640 10.1016/S0140-6736(02)11602-3 12457787 PMC3752775

[B10] Melchior M Moffitt TE Milne BJ Poulton R Caspi A Why do children from socioeconomically disadvantaged families suffer from poor health when they reach adulthood? A life- course study Am J Epidemiol 2007 166 966 974 10.1093/aje/kwm155 17641151 PMC2491970

[B11] Roberts RE Roberts Ramsay C Xing Y Rates of DM-IV psyhicatric disorders among adolescents in a large metropolitan area J Psy Res 2007 41 959 967 10.1016/j.jpsychires.2006.09.006 PMC2736593 17107689

[B12] Glasskock DJ Andersen J Labriola M Rasmussen K Hansen CD Can negative life events and coping style help explain socioeconomic differences in perceived stress among adolescents? A cross-sectional study based on the West Jutland cohort study BMC Public Health 2013 13 532 10.1186/1471-2458-13-532 23724872 PMC3679909

[B13] Currie C Molcho M Boyce W Holstein B Torsheim T Richter M Researching health inequalities in adolescents: the development of the Health Behaviour in School-aged Children (HBSC) family affluence scale Soc Sci Med 2008 66 1429 1436 10.1016/j.socscimed.2007.11.024 18179852

[B14] Chen E Martin AD Matthews KA Socioeconomic status and health: Do gradients differ within childhood and adolescence? Soc Sci Med 2006 62 2161 2170 10.1016/j.socscimed.2005.08.054 16213644

[B15] Lien N Friestad C Klepp K-I Adolescents’ proxy reports of parents’ socioeconomic status: How valid are they? J Epidemiol Comm Health 2001 55 731 737 10.1136/jech.55.10.731 PMC1731778 11553657

[B16] Goodman E The role of socioeconomic status gradients in explaining differences in US adolescents’ health Am J Public Health 1999 89 1522 1528 10.2105/AJPH.89.10.1522 10511834 PMC1508793

[B17] Jeynes WH The challenge of controlling for SES in social science and education research Educ Psy Rev 2002 14 205 221 10.1023/A:1014678822410

[B18] Kind P Dolan P Gudex C Williams A Variations in population health status: results from a United Kingdom national questionnaire survey BMJ 1998 316 736 741 10.1136/bmj.316.7133.736 9529408 PMC28477

[B19] Breidablick H-J Meland E Lydersen S Self-rated health in adolescence: a multifactorial composite Scand J Public Health 2008 36 12 20 10.1177/1403494807085306 18426780

[B20] Giannakopoulos G Mihas C Dimitrakaki C Tountas Y Family correlates of adolescents emotional/behavioural problems: evidence from a Greek school-based sample Acta Paediatr 2009 98 1319 1323 10.1111/j.1651-2227.2009.01314.x 19432840

[B21] Levin KA Currie C Muldoon J Mental well-being and subjective health of 11- to 15-year-old boys and girls in Scotland, 1994–2006 Eur J Public Health 2009 19 605 610 10.1093/eurpub/ckp046 19383842

[B22] Tobias M Kokaua J Gerritsen S Templeton R The health of children in sole-parent families in New Zealand: results of a population-based cross-sectional survey Aust N Z J Public Health 2010 34 274 280 10.1111/j.1753-6405.2010.00526.x 20618269

[B23] Kwan YK Ip WC Adolescent health in Hong Kong: disturbing socio-demographic correlates Soc Indicators Res 2009 91 259 268

[B24] Vingilis ER Wade TJ Seeley JS Predictors of adolescent self-rated health. Analysis of the National Population Health Survey Can J Public Health 2002 93 193 197 12050986 10.1007/BF03404999 PMC6979869

[B25] Vollebergh WAM Have M Dekovic M Oosterwegel A Pels T Veenstra R de Winter A Ormel H Verhulst F Mental health in immigrant children in the Netherlands Soc Psychiatry Psychiatr Epidemiol 2005 40 489 496 10.1007/s00127-005-0906-1 16003599

[B26] Pantzer K Rajmil L Tebé C Codina F Serra-Sutton V Ferrer M Ravens-Sieberer U Simeoni M-C Alonso J Health related qulity of life in immigrants and native school aged adolescents in Spain J Epidemiol Comm Health 2006 60 694 698 10.1136/jech.2005.044073 PMC2588083 16840759

[B27] Murad Darwish S Joung IMA van Lenthe FJ Bengi-Arslan L Crijnen AAM Predictors of self-reported problem behaviours in Turkish immigrant and Dutch adolescents in the Netherlands J Child Psychol and Psych 2003 44 412 423 10.1111/1469-7610.00131 12635970

[B28] Focus, Swedish news magazine http://www.fokus.se

[B29] Statistics Sweden Statistical Database http://www.scb.se

[B30] Bhatia S Jenney MEM Wu E Bogue MK Rockwood TH Feusner JH Friedman DL Robison LL Kane RL The Minneapolis-Manchester quality of life instrument: reliability and validity of the youth form J Pediatr 2004 145 39 46 10.1016/j.jpeds.2004.02.034 15238904

[B31] Bhatia S Jenney MEM Wu E Bogue MK Rockwood TH Feusner JH Friedman DL Robison LL Kane RL The Minneapolis-Manchester quality of life instrument: reliability and validity of the adolescent form J Clin Oncol 2002 20 4692 4698 10.1200/JCO.2002.05.103 12488415

[B32] Einberg E-L Kadrija I Brunt D Nygren J Svedberg P Psychometric evaluation of a Swedish version of Minneapolis-Manchester quality of life-youth form and adolescent form Health Qual Life Outcomes 2013 11 1 8 10.1186/1477-7525-11-1 23656858 PMC3651703

[B33] Rice N Leyland A Multilevel models: applications to health data J Health Serv Res Policy 1996 1 154 164 10180862 10.1177/135581969600100307

[B34] Nielsen L Vinther-Larsen M Nielsen NR Grönbäck M Stress Bland Unge 2007 Copenhagen: Sundhedsstyrelsen

[B35] Halldorsson M Kunst AE Köhler L Mackenbach JP Socioeconomic inequalities in the health of children and adolescents. A comparative study of the fie Nordic countries Eur J Public Health 2000 10 281 288 10.1093/eurpub/10.4.281

[B36] Goodman E Huang B Shafer-Kalkhoff T Adler NE Perceived socioeconomic status: a new type of identity that influences adolescents’ self-rated health J Adolesc Health 2007 41 479 487 10.1016/j.jadohealth.2007.05.020 17950168 PMC2204090

[B37] Amato PR Children of divorce in the 1990s: an update of the Amato and Keith (1991) meta-analysis J Fam Psychol 2001 15 355 370 11584788 10.1037//0893-3200.15.3.355

[B38] van Leeuwen N Rodgers R Régner I Chabrol H The role of acculturation in suicidal ideation among second-generation immigrant adolescents in France Transcult Psy 2010 47 812 832 10.1177/1363461510382154 21088105

[B39] Stevens GWJM Vollebergh WAM Mental health in migrant children J Child Psychol Psychiatry 2008 49 276 294 10.1111/j.1469-7610.2007.01848.x 18081765

[B40] Dimitrova R Children’s social relationships in the Northern Italian school context: evidence for the immigrant paradox JMIS 2011 16 478 491

[B41] Carlsund Å Eriksson U Sellström E Shared physical custody after family split-up: implications for health and well-being in Swedish schoolchildren Acta Paediatr 2013 102 318 323 10.1111/apa.12110 23190407

[B42] Hoffmann JP Family structure, community context, and adolescent problem behaviors J Youth Adolesc 2006 35 867 880 10.1007/s10964-006-9078-x

[B43] Vousoura E Verdeli H Warner V Wickramaratne P Baily C Parental divorce, familial risk for depression, and psychopathology in offspring: a three-generation study J Child Fam Stud 2012 21 718 725 10.1007/s10826-011-9523-7 PMC5644394 29051699

[B44] Güngör D Bornstein MH Gender, development, values, adaptation, and discrimination in acculturating adolescents: the case of turk heritage youth born and living in Belgium Sex Roles 2009 60 537 548 10.1007/s11199-008-9531-2

[B45] Jung S-H Tsakos G Sheiham A Ryu J-I Watt RG Socio-economic status and oral health-related behaviours in Korean adolescents Soc Sci Med 2010 70 1780 1788 10.1016/j.socscimed.2010.02.022 20359807

